# Investigation on the Fatigue and Rutting Behavior of Asphalt Binder Containing Compound Warm Mixing Agent

**DOI:** 10.3390/ma19102136

**Published:** 2026-05-19

**Authors:** Qinghong Fu, Tingting Chang, Qing Yang, Nong Zhang, Ziyang Huang, Keyu Yu, Qi Li

**Affiliations:** College of Engineering, Zhejiang Normal University, Jinhua 321004, China; shandafqh@163.com (Q.F.); changtingting@zjnu.edu.cn (T.C.); zhangnong2000@zjnu.edu.cn (N.Z.); 2799789365@zjnu.edu.cn (Z.H.); 2162664504@zjnu.cn (K.Y.); zjwclq@zjnu.edu.cn (Q.L.)

**Keywords:** composite warm-mix additive, warm-mix asphalt binder, optimum dosage, viscosity, rheological properties

## Abstract

**Highlights:**

- Develop a novel organic viscosity-reducing warm mix asphalt additive (PNSK).
- Determine the optimal PNSK dosage at 11% by balancing different properties.
- Demonstrate superior fatigue resistance and lower strain sensitivity of PWMA through LAS.
- Characterize the stress-temperature sensitivity and deformation behavior using MSCR.

**Abstract:**

A composite warm-mix additive (PNSK) was developed to improve asphalt workability by reducing viscosity while maintaining rheological performance at both high and low temperatures. The warm-mix asphalt binders (PWMA) were analyzed using an integrated approach combining conventional property tests with rheological analysis. Results showed that penetration, softening point, and ductility improved. The viscosity-reduction effect was enhanced with increasing PNSK dosage, yet the benefit plateaued beyond 11% content. Additionally, the adhesion strength between asphalt and aggregate began to decrease after 11% dosage, with 12% serving as the critical threshold for adhesion deterioration. Consequently, the optimal dosage was determined to be 11% based on comprehensive consideration of all factors. LAS results demonstrated that 11%PWMA exhibited lower strain sensitivity and superior fatigue resistance at low-to-intermediate temperatures, with fatigue life increasing by nearly an order of magnitude under low strain at 20 °C. MSCR results revealed that under low stress, 11%PWMA exhibited significantly lower non-recoverable creep compliance (*J_nr_*) and higher percent recovery (*R*) than the 70^#^, especially in the high-temperature range (54–66 °C), demonstrating superior resistance to permanent deformation. However, 11%PWMA exhibited temperature-strain sensitivity characteristics under high-temperature, high-strain conditions, representing an inherent characteristic of WMA technology.

## 1. Introduction

Amid the global push for green development, traditional hot-mix asphalt (HMA) faces criticism for high energy use and pollution [1]. Warm-mix asphalt (WMA) has thus emerged as a key research focus. By lowering production temperatures by 15–30 °C [2], WMA reduces energy consumption and emissions, enabling low-carbon construction [3]. It also improves working conditions while matching HMA performance. This technology supports sustainable development in road construction without compromising pavement quality.

WMA technology has evolved into three main categories: organic viscosity-reduction, surfactant-based technologies, and foaming processes [4]. Organic viscosity-reducing additives lower binder viscosity, enabling mixing and compaction at reduced temperatures. These additives typically consist of synthetic organic waxes, such as Fischer-Tropsch, amide, and montan waxes [5,6]. A well-known example is Sasobit, a granular aliphatic hydrocarbon produced via the Fischer-Tropsch process with a melting range of 90–120 °C. Above this range, Sasobit rapidly melts, reducing binder viscosity and improving aggregate coating, thus allowing lower production and paving temperatures [7].

Current warm-mix technologies mainly rely on single-component additives. While effective in reducing viscosity and energy consumption, this approach faces limitations—particularly the trade-off between high-temperature stability and low-temperature crack resistance, along with high overall costs [8,9]. For example, Li et al. found that Sasobit improves rutting and aging resistance but reduces low-temperature performance. Similarly, additives like XT-W3 and Evotherm lower high-temperature viscosity and enhance low-temperature properties, yet slightly weaken deformation resistance [10]. Quantitatively, Sasobit, XT-W3, and Evotherm lower the mixing temperature by 20–40 °C. At 135 °C, viscosity reduction ranges are 21–32% (Sasobit), 9–14% (XT-W3), and 16–30% (Evotherm). At 64 °C, Sas obit increases |G*|/sin*δ* by 83–213%, while XT-W3 and Evotherm show smaller gains (10–35% and 24–48%). However, each has inherent limitations. Sasobit compromises low-temperature cracking resistance. XT-W3 offers limited viscosity reduction and modest high-temperature gain. Evotherm provides only moderate performance improvements. To overcome such trade-offs, researchers have explored multi-component strategies. Ning developed a synergistic viscosity-reducing agent that achieved more balanced high- and low-temperature performance [11]. Dong et al. reported that Acmp softens asphalt during mixing, improving low-temperature flexibility, while partial volatilization of light components limits damage to the high-temperature skeleton, helping maintain rutting resistance [12]. Sedagha et al. combined Sasobit with nano-montmorillonite K10 and found improved rheological properties and better low-temperature cracking resistance [13]. Duan et al. further identified that waxes with molecular weights between 1300 and 1500 g/mol significantly reduce viscosity and enhance rutting resistance, offering a quantitative basis for optimizing warm-mix additives [14].

The dosage of a warm-mix additive is not a matter of simple addition. Instead, an optimal window frequently acts as the critical threshold that dictates the overall binder performance. Consequently, accurate identification of this “optimum dosage” has become the primary issue in performance tuning [15,16]. Liang X et al. reported that 3% Sasobit or EC120 improves high-temperature stability but sacrifices low-temperature cracking resistance and fatigue life, whereas 1% Evotherm delivered the best compromise among high- and low-temperature properties and fatigue durability, a 4% dosage of water-based foaming agent, by contrast, exerted only marginal influence [17]. These results underscore that precise dosage optimization is the key to proper control. Wang et al. integrated high-temperature rheological indices (rutting factor |G*|/sin*δ* and non-recoverable creep compliance *J_nr_*) with low-temperature parameters (creep stiffness *S* and *m*-value) to determine the optimum contents: 3% for Sasobit and 1.0% for both Evotherm M-1 and YDWB-1, at which the binder achieves the best balance between high-temperature stability and low-temperature cracking resistance [18].

The rheological behavior of asphalt binder is the decisive metric for its performance under various loading and temperature regimes [19]. The Strategic Highway Research Program (SHRP) Performance Grade (PG) framework was the first to link binder rheology to field performance, establishing specification limits that reflect true engineering demands [20]. Zhang et al. employed Multiple Stress Creep and Recovery (MSCR) and Linear Amplitude Sweep (LAS) tests to quantify cracking resistance from high- to intermediate-temperature domains: MSCR showed that wax inhibitors (WIs) reduce non-recoverable creep compliance (*J_nr_*) and boost percent recovery, implying enhanced rutting resistance, whereas LAS revealed higher fatigue-failure strain and prolonged fatigue life with WI modification [21]. Bhat et al. adopted MSCR double-stress cycles to extract *J_nr_* and recovery (*R*), adopting *J_nr_* < 0.5 kPa^−1^ as the threshold for extreme-traffic suitability. LAS-derived damage parameters *C*_1_, *C*_2_ and cycles to failure *N_f_* were used to quantify the synergistic improvement conferred by nano-Al_2_O_3_ and warm-mix additives, with higher *N_f_* signifying superior fatigue endurance [22]. Collective evidence indicates that different warm-mix additives impose distinct signatures on binder rheology. Wasiuddin et al. compared Sasobit and Aspha-Min in PG 64-22 and PG 70-28 binders, demonstrating that Sasobit markedly elevates the rutting factor |G*|/sin*δ* and reduces rut depth, the two variables exhibiting strong positive correlation [23]. Morea et al. compared conventional and polymer-modified binders from HMA and WMA pavements, finding polymer-modified systems sensitive to WMA chemistry, while conventional grades remain unaffected [24]. Systematic rheological mapping, therefore constitutes an effective tool for predicting the field performance of WMA [25]. Comparing different WMA additives and interpreting their rheological effects provides essential theoretical guidance for optimizing WMA technology applications.

To overcome the limitations of organic viscosity-reducing warm-mix additives, a composite additive (PNSK) was developed. Through synergistic effects, it enhances both high-temperature rutting resistance and low-temperature cracking resistance, avoiding the trade-offs of single-component modifications. Macroscopic tests (penetration, ductility, softening point) were conducted to determine the optimal dosage. LAS and MSCR tests were then used to evaluate creep, recovery, and long-term durability.

## 2. Materials and Sample Preparation

### 2.1. Raw Materials

#### 2.1.1. Asphalt

The 70^#^ base asphalt binder used in this study was supplied by Sinopec Zhenhai Refining & Chemical Company (Ningbo, China). Its performance grade (PG) is PG 64-22. The binder was selected for its stable performance and widespread application in pavement engineering. To ensure the reliability of subsequent experiments, the basic physical properties were systematically evaluated in accordance with ASTM standards.

#### 2.1.2. Constituents of Warm Mix Additive

The composite warm-mix additive (PNSK) was developed in-house and formulated as a composite system composed primarily of synthetic waxes and oil-based substances, supplemented by an anti-stripping agent and synergists. Component 1 is Synthetic wax (-(C_2_H_4_)_n_-), which leverages its long-chain alkyl structure to serve as the core viscosity reducer, significantly lowering mixing and compaction temperatures. Component 2 is Plasticizer (C_n_H_2n−x_), which utilizes the penetration effect of its naphthenic and aromatic ring structures to weaken asphaltene–resin interactions and, synergistically with Synthetic wax, forms a composite viscosity-reduction system that reduces viscosity and improves low-temperature flexibility. Component 3 is Coupling agent (R-Si-X_3_), which creates chemical bonds at the aggregate–binder interface through the silyl and organic functional groups, enhancing adhesion and moisture resistance. Component 4 is Antioxidant compatibilizer (-(C_2_H_6_OSi)_n_-), which reduces internal friction and suppresses wax crystallization by the flexible siloxane backbone and hydrophobic methyl side groups, thereby improving processability. Its chemical inertness and film-forming ability also retard thermal-oxidative ageing, ensuring long-term storage and field stability. Through the synergistic effects of physical blending and molecular compatibility, PNSK simultaneously improves high-temperature rutting resistance and low-temperature cracking resistance, overcoming the inherent limitations of single-component modifiers [26]. Relevant technical properties of all materials are given in Table 1. To guarantee consistency, all materials were taken from the same production batch.

### 2.2. Sample Preparation

In this study, a precision-temperature-controlled (±1 °C) electric heating mantle coupled with a mechanical stirring system was employed to synthesize the PNSK. First, Synthetic wax, Plasticizer, Coupling agent, and Antioxidant compatibilizer were accurately weighed beforehand and mixed in a ratio of 5:3:1:1, then introduced into the reactor vessel. The reaction mixture was then placed in the temperature-controlled heating mantle and heated. Meanwhile, a constant mechanical shear of 300 ± 10 r min^−1^ was applied. When the internal temperature reached 110 °C, the mixture was held isothermal for 30 min under continuous shearing to ensure complete melting of Synthetic wax and formation of a homogeneous phase with Plasticizer, yielding a pale yellow transparent liquid. Upon cooling to ambient temperature, the composite underwent a phase transition, solidifying into a pale-yellow solid with a regular crystalline texture. Long-term stability was verified by storing the product in a sealed container at room temperature for seven days. No phase separation, crystalline exudation, or turbidity change was observed, confirming excellent storage stability of the PNSK composite. For brevity in subsequent discussions, the WMA prepared herein is abbreviated as PWMA, where “PNSK” denotes the composite warm-mix additive and “a” its dosage (mass%) relative to the base binder. The list of abbreviations is provided before the References section.

**Figure 1:**
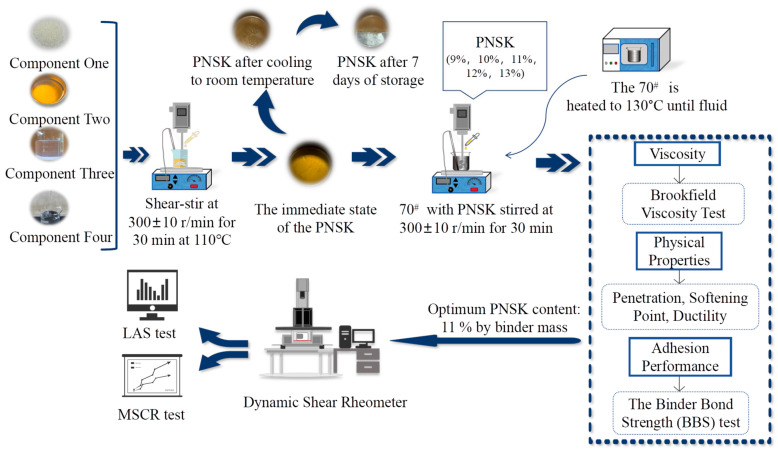
A graphical flowchart of experimental programs conducted.

To prepare PWMA, PNSK was added at mass fractions of 9%, 10%, 11%, 12%, and 13% to 300 g of the 70^#^. These dosages were selected based on a preliminary trial, which identified an effective working range for further evaluation. The blend was sheared at 300 r min^−1^ for 30 min in the same temperature-controlled heating mantle maintained at 110 °C, yielding the PWMA. A graphical flowchart of experimental programs conducted in this study is shown in Figure 1.

## 3. Experimental Design

### 3.1. Basic Physical Property Test

In this research, penetration (25 °C), softening point, and ductility (10 °C) were carried out to evaluate the physical properties of the prepared asphalt binder samples. These tests were conducted in accordance with the requirements of T0604, T0605, and T0606 test procedures in Standard Test Methods of Bitumen and Bituminous Mixtures for Highway Engineering (JTG 3410-2025) [27].

### 3.2. Brookfield Viscosity Test

According to ASTM D4402 [28], the DV2T Brookfield viscometer was used in this paper to test the viscosity of the asphalt binders. The rotation speed of the instrument was 50 rpm, and the test temperatures were 90 °C, 100 °C, 110 °C, 120 °C, and 130 °C, respectively. In this specification, (0.17 ± 0.02) Pa·s and (0.28 ± 0.03) Pa·s were taken as the mixing and compaction temperatures for asphalt mixtures, respectively [29]. Viscosity reduction across all test temperatures for each content was assessed through triplicate parallel tests at each temperature, using the ∆*V_ij_* and *VR_i_* (Viscosity Reduction) indicators from Equations (1) and (2).

(1) $$\Delta V_{ij}=V_{0j}-V_{i}$$

(2) $${VR}_{i}=\sum_{j-1}^{m}\Delta V_{ij}$$

where ${VR}_{i}$ is the sum of viscosity differences between WMA and 70^#^ at each temperature point for the ith addition content. *m* is the number of measured temperature points. $V_{0j}$ is the viscosity of 70^#^ at the jth temperature, and $V_{ij}$ is the viscosity of WMA at the ith addition content and jth temperature. In this paper, *m* is 5, *i* = 1, 2, 3, 4, 5, representing the content of 9%, 10%, 11%, 12%, and 13%, respectively. *j* = 1, 2, 3, 4, 5, representing the temperature of 90 °C, 100 °C, 110 °C, 120 °C, and 130 °C, respectively.

### 3.3. BBS Test

The Binder Bond Strength (BBS) test, as an important experimental method in the AASHTO standard for evaluating asphalt adhesion performance, can intuitively reveal the bonding characteristics between asphalt and aggregate. In this study, the PosiTest AT-A automatic pull-off adhesion tester (DeFelsko Corporation, New York, NY, USA) was used to conduct the BBS pull-off test. The procedure of the BBS pull-off test is as follows. First, a pull-off stub with a diameter of 20 mm, featuring a groove (0.8 mm thick) and an overflow hole, together with a basalt stone slab, was placed in an oven at 130 °C for 1 h to remove moisture from the pores of the stone slab. Second, a proper amount of asphalt in a fluid state was then dropped onto the stone slab. Then, the heated pull-off stub was placed over the asphalt, and a stone slab was placed on top of the stub to apply a certain pressure, ensuring that excess asphalt in the groove could flow out through the overflow hole. Finally, after cooling at room temperature for 4 h to develop bonding strength, the weight stone slab was removed, and the pull-off tests were performed. During the test, the pull-off rate was maintained at 0.7 MPa/s, and the temperature was kept at (25 ± 1) °C. To ensure data reliability, four replicate specimens were prepared for each asphalt binder. Outliers were identified using the ±2σ criterion (i.e., values deviating by more than two standard deviations from the mean were excluded). After outlier removal, the arithmetic mean of the valid measurements was adopted as the representative index of interfacial bond strength.

### 3.4. LAS Test

LAS test, grounded in the viscoelastic continuum damage (VECD) model and codified in AASHTO TP 101-14 [30], is an accelerated fatigue procedure. By DSR test, increasing shear amplitudes are applied to the specimen, and the resulting stress–strain data are analyzed within the continuum damage mechanics framework to predict the fatigue life and damage tolerance of the asphalt binders under various load levels. In this study, LAS tests were conducted using 8 mm parallel plates with a 2 mm gap at 20 °C, 25 °C, and 30 °C (low-to-moderate temperatures) to evaluate the influence of a warm-mix additive on the fatigue durability of the binder.

In this study, the prescribed protocol is implemented in two sequential stages. Step 1—Frequency sweep: A DSR applied a constant-amplitude oscillatory shear to the asphalt binder specimen while the frequency was automatically scanned from 0.2 Hz to 30 Hz at a fixed strain level of 0.1%. This built-in frequency-sweep routine furnished the linear-viscoelastic (LVE) material functions required for subsequent damage analysis. Step 2—Amplitude sweep: At a constant frequency of 10 Hz, the strain amplitude was increased linearly from an initial value of 0.1% to 30% at a rate of 1% s^−1^, delivering 3100 continuous load cycles. The monotonically rising cyclic strain accelerates fatigue micro-damage and enables a systematic evaluation of the binder’s fatigue resistance. Finally, the LVE functions obtained in Step 1 and the amplitude-sweep data recorded in Step 2 are combined to compute the fatigue-resistance parameter in accordance with the multistage approach proposed in [31]. The evolution of fatigue damage is quantified by the cumulative damage index *D*, defined in Equation (3).

(3) $$D(t)≅\sum_{i=1}^{N}{\left[\pi \gamma_{0}^{2}(C_{i-1}-C_{i})\right]}^{\frac{\alpha }{1+\alpha }}{\left(t_{i}-t_{i-1}\right)}^{\frac{1}{1+\alpha }}$$

where *C_t_* = G^*^(*t*)/G^*^, *C_t_* is the integrity parameter that is the applied strain, %, G^*^ is the complex shear modulus, *t* is time, and *α* is the coefficient, which is obtained using the following formula.

(4) $$\log(G^{*}\cos\delta )=\log G^{\prime }\left(\omega \right)=m\left(\log\omega \right)+b$$

(5) α=1+1/m

where *m* is the slope of the logarithmic curve between the storage modulus and the applied frequency, which is obtained from the fitting curve. The relationship between the integrity parameter *C* and the cumulative damage parameter *D* is established, as shown in Equation (6).

(6) $$C(t)=C_{0}-C_{1}{\left(D\right)}^{C_{2}}$$

where *C*_0_ = 1, *C*_1_ and *C*_2_ are fitting parameters obtained from the power-law linearization of Equation (7).

(7) $$\log(C_{0}-C(t))=\log\left(C_{1}\right)+C_{2}\cdot \log\left(D\right)$$

Fatigue damage is assumed to occur when the integrity parameter *D*(*t*) drops to 35%, at which point the damage level is quantified by *D_f_*, calculated from the following equation.

(8) $$D_{f}\;=(0.35){\left(\frac{C_{0}}{C_{1}}\right)}^{1/C_{2}}$$

The parameters *A*_35_ and *B* of the asphalt binder fatigue-performance model are calculated using Equation (9).

(9) $$A_{35}=\frac{{f(D_{f})}^{k}}{{k(\pi I_{D}C_{1}C_{2})}^{\alpha }}$$

In this equation, *f* is the loading frequency, *f* = 10 Hz, and *k* = 1 + *α*(1 − *C*_2_), *B* = 2*α*. The fatigue-performance parameter of the asphalt binder is calculated using Equation (10).

(10) $$N_{f}=A_{35}{\left(\gamma_{\max}\right)}^{B}$$

where $\gamma_{\max}$ denotes the maximum allowable strain of the asphalt binder.

### 3.5. MSCR Test

**Figure 2:**
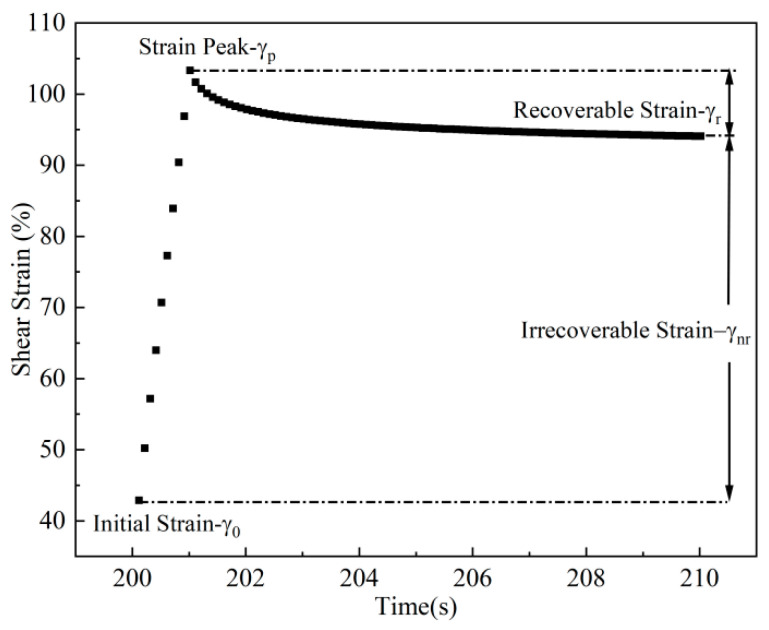
Schematic of a single repeated-creep loading cycle.

MSCR test was conducted in accordance with AASHTO TP 350-14 [32]. In this study, two stress levels—0.1 kPa and 3.2 kPa—were selected to simulate the creep response under different traffic volumes. The test was performed under a 1 s loading followed by a 9 s recovery cycle, repeated multiple times to capture the strain development within a single creep–recovery period [33]. The results are presented in Figure 2.

In general, the repeated-creep performance of an asphalt binder is evaluated by means of the strain-recovery ratio *R* and the non-recoverable creep compliance *J_nr_*. The corresponding calculations are given in Equations (11) and (12) [34].

Strain-Recovery Ratio *R*:

(11) $$R=\frac{\gamma_{r}}{\gamma_{p}-\gamma_{0}}\times 100\%$$

Non-recoverable Creep Compliance *J_nr_*:

(12) $$J_{\mathrm{nr}}=\frac{\gamma_{\mathrm{nr}}}{\tau }\times 100\%$$

where $\gamma_{0}$ denotes the initial strain, %, $\gamma_{p}$ denotes the peak strain, %, $\gamma_{r}$ denotes the recoverable strain, %, $\gamma_{\mathrm{nr}}$ denotes the non-recoverable strain, %, *τ* denotes the creep loading stress, kPa.

Under the stress levels of 0.1 kPa and 3.2 kPa, the recovery rates for the first creep–recovery cycle are calculated using Equations (13) and (14), respectively.

(13) $$R_{r}(0.1,N)=\frac{\gamma_{r}}{\gamma_{p}-\gamma_{0}}\times 100\%,N=(1,10)$$

(14) $$R_{r}(3.2,N)=\frac{\gamma_{r}}{\gamma_{p}-\gamma_{0}}\times 100\%,N=(1,10)$$

Under stress levels of 0.1 kPa and 3.2 kPa, the average creep-recovery rate after ten creep–recovery cycles is expressed by Equations (15) and (16), respectively.

(15) $$R_{0.1}=\frac{\sum_{N}^{10}R_{r}(0.1,N)}{10},N=(1,10)$$

(16) $$R_{3.2}=\frac{\sum_{N}^{10}R_{r}(3.2,N)}{10},N=(1,10)$$

Under stress levels of 0.1 kPa and 3.2 kPa, the non-recoverable deformation after ten creep–recovery cycles is given by Equations (17) and (18), respectively.

(17) $$J_{\mathrm{nr}}(0.1,10)=\frac{\gamma_{\mathrm{nr}}}{10},N=(1,10)$$

(18) $$J_{\mathrm{nr}}(3.2,10)=\frac{\gamma_{\mathrm{nr}}}{3200},N=(1,10)$$

Under stress levels of 0.1 kPa and 3.2 kPa, the average non-recoverable deformation over ten creep–recovery cycles is given by Equations (19) and (20), respectively.

(19) $$J_{\mathrm{nr}0.1}=\frac{\sum_{N}^{10}J_{\mathrm{nr}}(0.1,N)}{10},N=(1,10)$$

(20) $$J_{\mathrm{nr}3.2}=\frac{\sum_{N}^{10}J_{\mathrm{nr}}(3.2,N)}{10},N=(1,10)$$

The parameters *R_diff_* and *J_nr-diff_* effectively characterize the stress-sensitivity of the asphalt binders. Their magnitudes are positively correlated with the degree to which the binder responds to changes in stress level [35]. The corresponding calculations are provided in Equations (21) and (22).

(21) $$R_{\mathrm{diff}}=\frac{R_{0.1}-R_{3.2}}{R_{0.1}},N=(1,10)$$

(22) $$J_{\mathrm{nr}-\mathrm{diff}}=\frac{J_{3.2}-J_{0.1}}{J_{0.1}},N=(1,10)$$

## 4. Results and Discussion

### 4.1. Physical Characteristics

To evaluate the influence of the PNSK on the traditional physical properties of the asphalt binders, the traditional physical characteristics tests were conducted on PWMA containing 9%, 10%, 11%, 12%, and 13% PNSK by mass. The results are presented in Table 2.

The penetration, softening point, and ductility test results of PWMA are summarized in Table 2. As illustrated in Table 2, PWMA exhibits a stable, linear increasing trend in its properties. With the dosage increasing from 9% to 13%, the penetration increment progressively increases from 27.1% to 36.6%, the softening point increase rate synchronously rises from 25.1% to 35.5%, and the ductility demonstrates continuous improvement, demonstrating that PWMA simultaneously enhances the deformation capacity and high-temperature stability of asphalt [36]. These results serve as a preliminary assessment, while a more comprehensive evaluation of fatigue resistance and rutting behavior based on LAS and MSCR analyses will be presented in subsequent sections. Notably, these improvement effects are significantly amplified at higher PWMA contents. This performance enhancement mechanism can be attributed to the saturated cyclic carbon chain structure of Plasticizer in PWMA, which likely interacts with the 70^#^, enhancing and crosslinking polymer chains while reducing the stiffness and strength of asphalt, which macroscopically manifests as decreased viscosity and increased penetration. However, using Plasticizer alone would cause a decrease in the softening point, which is compensated by the concurrent presence of Synthetic wax that forms a three-dimensional network structure through crystallization to enhance high-temperature stability. Consequently, PWMA significantly improves low-temperature ductility, with gradual enhancement observed as dosage increases. This gain is attributed to the Plasticizer’s suppression of the Synthetic wax’s crystallinity, thereby expanding the plastic deformation zone. The synergistic effect of the two components thus yields a binder with simultaneously enhanced low-temperature crack resistance and high-temperature stability.

### 4.2. Evaluation of Viscosity-Temperature Characteristics

#### 4.2.1. Impact of PNSK Content on Viscosity Reduction

**Figure 3:**
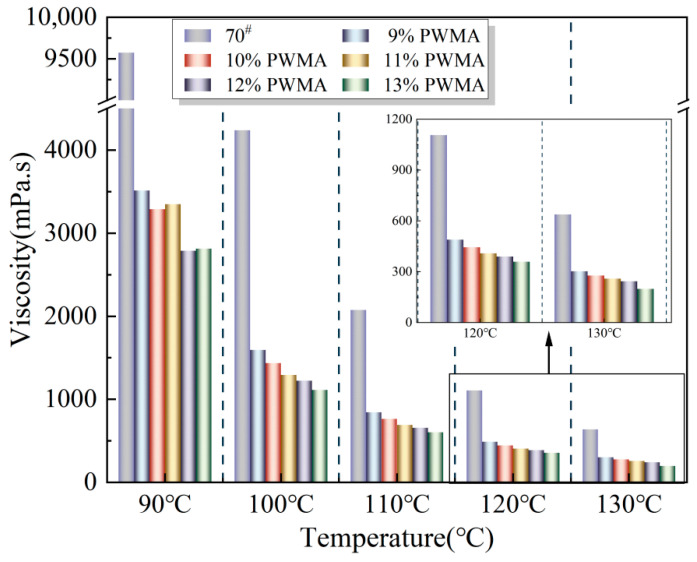
Viscosity of different asphalt binders at different temperatures.

Rotational viscosity tests were conducted on 70^#^ and PWMA containing various PNSK dosages. The Brookfield viscosities measured at multiple temperatures are summarised in Figure 3. At every test temperature, binder viscosity decreases monotonically with increasing PNSK content. This reduction is ascribed to the synergistic action of Synthetic wax and Plasticizer in the PNSK additive. Synthetic wax disperses throughout the binder as micro-scale lubricating particles that diminish internal friction, whereas the saturated cyclic carbon chains of Plasticizer intercalate between asphaltene molecules, enlarge inter-molecular distances, and weaken associative forces at the molecular level. Additionally, viscosity exhibits a strong negative correlation with temperature, a trend consistent with expected asphalt rheology: elevated thermal agitation and expanded molecular spacing at higher temperatures jointly reduce inter-molecular attraction and thus lower viscosity.

**Figure 4:**
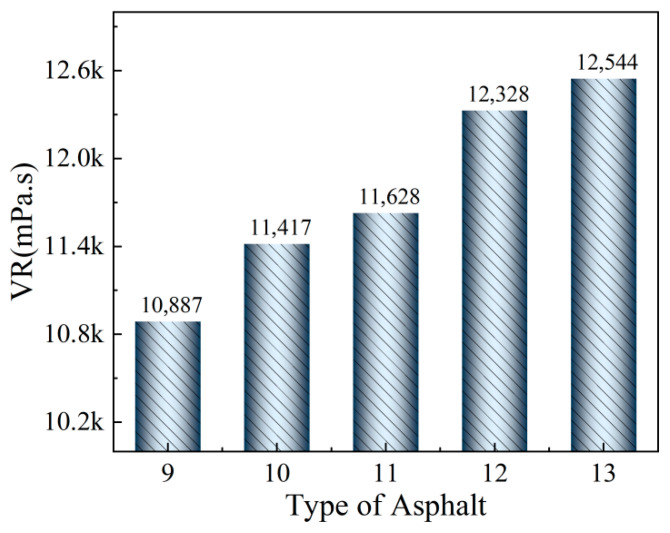
VR values of different asphalt binders with different content of PNSK.

The VR values of the asphalt binders were calculated and are presented in Figure 4, showing a significant increase that indicates a stronger viscosity-reducing effect at higher dosages [37]. As the PNSK content rises from 9% to 13%, VR continues to grow, but with a diminishing increment, beyond 11%, the marginal gain shrinks by ~38% due to the simultaneous approach to limiting levels of crystallization of Synthetic wax, dilution of Plasticizer and Coupling agent, and surface coverage by Antioxidant compatibilizer. Consequently, 11% PNSK achieves 94% of the maximum viscosity reduction while saving 2 wt% of the additive, constituting the economical optimum. Further dosage offers negligible rheological benefit and may even raise low-temperature stiffness, compromising thermal-cracking resistance.

#### 4.2.2. Temperature Sensitivity of the Asphalt Binders

**Figure 5:**
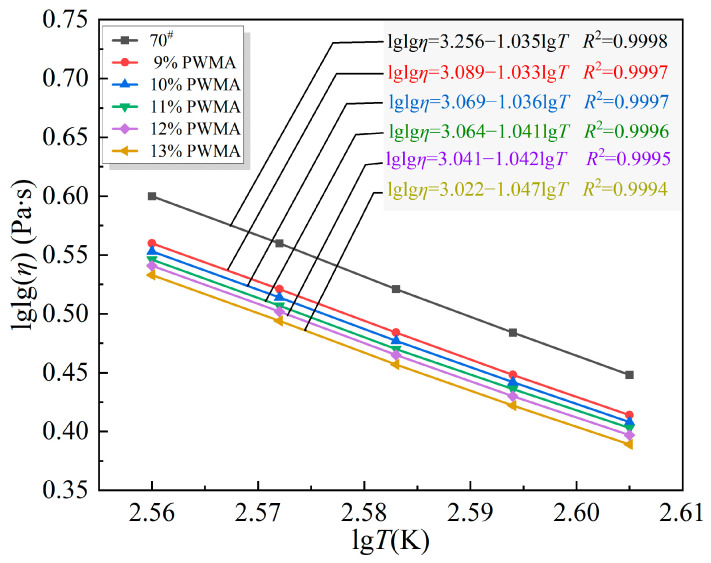
Viscosity–temperature curve of different asphalt binders.

Based on the test results in Figure 5, the influence of varying PNSK dosage on the viscosity characteristics of the binder was analyzed, and the corresponding viscosity–temperature curves were constructed. The curves were then fitted using the Saal equation. As illustrated in Figure 5, a linear relationship between the logarithm of temperature and the double logarithm of viscosity for the WMA samples satisfies the Saal formula (Equation (23)).

(23) lglg(η)=A−Blg(T+273.13)

where η denotes the asphalt binder viscosity, Pa·s, *T* denotes the temperature, °C, *A* and *B* denote the regression coefficients.

Parameter *B* reflects the temperature sensitivity of the binder. A higher *A* value indicates a stronger dependence of viscosity on temperature. The curves were fitted with Equation (23), and the results are displayed in Figure 5.

Figure 5 clearly shows that, as the temperature continuously increases, the Brookfield viscosity of both the 70^#^ and the PWMA decreases monotonically. This observation is consistent with the fundamental temperature-dependent rheology of the asphalt binders: elevated thermal agitation intensifies molecular motion, weakens intermolecular interactions, and consequently reduces viscosity while enhancing flowability.

In Equation (21), *A* is the intercept of the Saal model and sets the viscosity baseline at any given temperature, and a smaller *A* means a lower viscosity. *B* is the slope that gauges temperature sensitivity, and a higher *B* gives a faster viscosity drop as temperature rises. Figure 5 shows that 70^#^ has a smaller *B* and a larger *A* than any PWMA. Moreover, *B* increases monotonically while *A* decreases monotonically with PNSK dosage, so the PWMA is far less viscous than the 70^#^. This is ascribed to the combined action of Plasticizer and Coupling agent, which dilute the binder through their high free-volume expansion, Synthetic wax that instantaneously enlarges free volume upon melting, and the trace amount of Antioxidant compatibilizer that disperses asphaltene nuclei. Together, these effects render the binder markedly temperature-sensitive, a conclusion that agrees with the subsequent rheological analysis of the PWMA [38].

Taking 120 °C as an example, the viscosity values are 488.9 mPa·s at 9% PNSK and 358.3 mPa·s at 13% PNSK. These data demonstrate that the incorporation of PNSK effectively reduces the viscosity of the asphalt binders and markedly improves their flow behavior. Moreover, the viscosity-reducing benefit becomes more pronounced as the PNSK dosage increases. At every tested temperature, the Brookfield viscosity of the PWMA binders was consistently lower than that of the 70^#^. For instance, at 90 °C, the base binder registered 9575 mPa·s, whereas the binder containing the maximum 13% PNSK dosage reached only 2813 mPa·s. This pronounced reduction confirms that PNSK markedly decreases the asphalt binders’ viscosity, enabling the modified binder to achieve, at a considerably lower temperature, a flowability comparable to that of the 70^#^ at a higher temperature, thereby facilitating WMA concrete placement under cool-weather conditions.

The temperature range yielding a Brookfield viscosity of 0.17 ± 0.02 Pa·s was defined as the mixing window, while the range corresponding to 0.28 ± 0.03 Pa·s was taken as the compaction window. Based on the resulting viscosity–temperature curves and the magnitude of the temperature reduction induced by PNSK, the resultant construction temperatures are detailed in Table 3.

Table 3 reveals that the cooling benefit of PWMA intensifies progressively with increasing PNSK dosage, indicating a clear positive correlation between additive content and temperature reduction. However, the temperature reductions for 11% and 12% PWMA are 20 °C and 21 °C, respectively—a marginal difference of merely 1 °C. This minimal discrepancy indicates that additional gains in cooling efficiency become limited once PNSK dosage exceeds 11%.

### 4.3. Adhesion Performance Analysis of the Asphalt Binders

As shown in Table 4, the 70^#^ exhibits a pull-off strength of 3.05 MPa. Introducing PNSK systematically reduces this value in a non-linear manner. Within the 9–11 wt% dosage window, the strength only declines marginally (2.15 → 2.04 MPa). This plateau is attributed to Coupling agent, which forms a covalent bridge—anchoring to surface hydroxyls of the aggregate and to reactive functions of the asphalt binder—thereby retarding moisture ingress and moderating the strength loss. At 12 wt%, the strength drops abruptly to 1.24 MPa (39% decrease relative to 11 wt%), marking the critical threshold beyond which adhesion deteriorates sharply [39]. Further increasing the dosage to 13 wt% yields a strength of only 0.99 MPa (< 33% of the 70^#^). This 39–67% reduction in pull-off strength at 12–13% PNSK dosage directly suggests a critical risk of moisture-induced damage in pavements. Such a significant loss in pull-off strength would increase the susceptibility of the asphalt-aggregate interface to stripping under the combined action of water ingress and repetitive traffic loading, potentially leading to premature pavement deterioration [40]. Therefore, the 11 wt% threshold identified here serves as an indicator of binder-aggregate adhesion. However, it should be noted that these findings are based solely on binder-aggregate pull-off tests, and tests for mixtures will be performed in future research.

### 4.4. Determination of the Optimum PNSK Content

**Figure 6:**
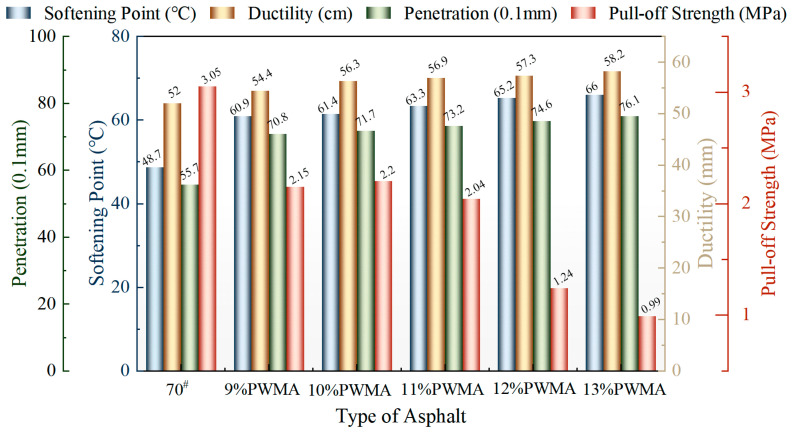
Performance comparison of different asphalt binders.

PNSK dosage substantially influences the softening point, penetration, and pull-off strength, as illustrated in Figure 6.

As illustrated in Figure 6, the 11%PWMA achieves an optimal equilibrium among softening point enhancement, interfacial adhesion, and temperature sensitivity. At this dosage, the softening point reaches 63.3 °C, representing a 30.0% increase compared with 70^#^ and signifying substantial improvement in high-temperature rutting resistance. Furthermore, the cooling effect of PWMA intensifies progressively with the increase in PNSK content. However, the temperature reductions for 11% and 12% PWMA are 20 °C and 21 °C, respectively—a marginal difference of merely 1 °C. This minimal discrepancy indicates that additional gains in cooling efficiency become limited once PNSK dosage exceeds 11%. Concurrently, the pull-off strength exhibits a gradual decline with rising PNSK content, precipitously dropping to 1.24 MPa at 12% dosage (a reduction of 39.2%) when the content surpasses 11%, thereby jeopardizing resistance to moisture-induced damage. Although the pull-off strength of 111%PWMA(2.04 MPa) is lower than that of 70^#^ (3.05 MPa), it remains within the engineering-acceptable threshold, suggesting low risk of interfacial debonding [41]. Consequently, 11% is established as the optimal PNSK dosage, maximizing high-temperature stability while retaining sufficient adhesive properties and minimizing process energy consumption without incurring disproportionate performance losses.

### 4.5. Fatigue Performance of the Asphalt Binders

#### 4.5.1. Stress–Strain Curve Analysis

**Figure 7:**
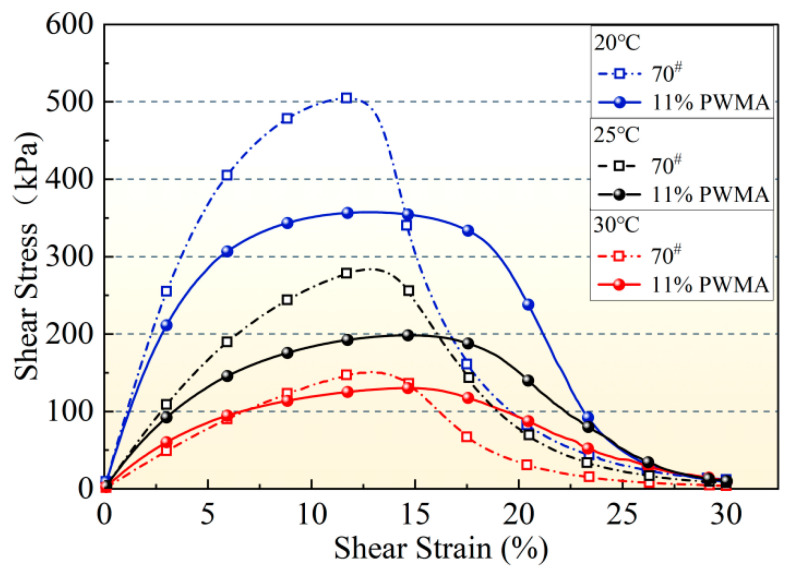
Stress–strain curves of different asphalt binders at 20 °C, 25 °C and 30 °C.

To investigate the effect of the PNSK on the fatigue resistance of asphalt binders, LAS tests were conducted on binders at low-to-moderate temperatures of 20 °C, 25 °C, and 30 °C. The results are presented in Figure 7. At all temperatures, both binders exhibit the same non-linear signature: shear stress increases with strain to a distinct peak and then decreases as damage accumulates beyond the critical strain, producing a clear inflection point.

11%PWMA displays a gentler pre-peak slope and a lower peak stress than the 70^#^, indicating reduced strain sensitivity. The additive widens the peak zone, allowing the material to sustain a relatively stable stress over a broader strain interval before macro-cracking initiates. In contrast, the 70^#^ shows a steeper ascent and a narrower peak, reflecting higher initial stiffness but more abrupt failure once the elastic limit is exceeded. Consequently, 11%PWMA possesses superior fatigue durability under repeated loading [42].

Raising the temperature from 20 °C to 30 °C progressively lowers the peak stress and reduces the post-peak slope for both materials, evidencing thermally accelerated molecular mobility and weakened intermolecular cohesion. Across the entire temperature range, 11%PWMA maintains a lower stress level and a wider peak-strain bandwidth, confirming its enhanced strain tolerance and fatigue life.

#### 4.5.2. Fatigue Damage Curve Analysis

**Figure 8:**
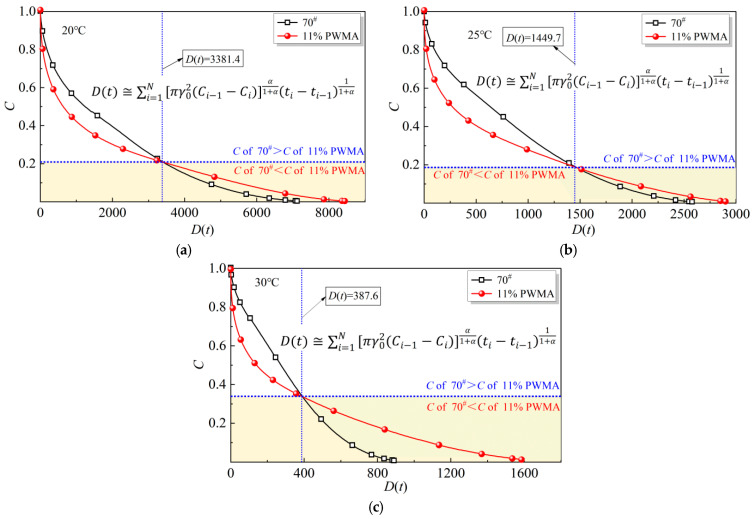
Fatigue-damage curves of different asphalt binders at (**a**) 20 °C, (**b**) 25 °C, and (**c**) 30 °C.

The damage characteristic curves constructed with the VECD model are presented in Figure 8, where *D*(*t*) denotes the accumulated fatigue-damage parameter. The ordinate *C* reflects the integrity of the asphalt binder specimen: a value of 1 corresponds to the intact (undamaged) state, whereas 0 indicates complete damage. For a given cumulative damage level, a higher *C* shows a superior fatigue resistance [43].

Figure 8 reveals that the 70^#^ experiences a relatively slow damage growth during the initial loading phase, attributable to its stable micro-structure that preserves molecular integrity and retards the accumulation of the damage parameter [44]. Once the critical damage threshold is reached, however, the curve drops sharply toward zero, signifying rapid internal deterioration and an accelerated loss of load-bearing capacity. This pattern demonstrates the binder’s limited ability to resist crack propagation after damage initiation. By examining the key nodal values of *D*(*t*) = 3381.4 at 20 °C, *D*(*t*) = 1449.7 at 25 °C, and *D*(*t*) = 387.6 at 30 °C, it is evident that the 11%PWMA experiences a relatively high damage rate at the very beginning of fatigue loading. However, as cumulative damage continues to increase, the damage-growth rate decreases markedly and remains consistently lower than that of the 70^#^. This behaviour demonstrates the progressive-damage characteristic of the 11%PWMA: although early flaws develop rapidly, the rate of subsequent degradation diminishes progressively with time, underscoring the material’s enhanced durability. This beneficial response is believed to originate from (i) Antioxidant compatibilizer of PNSK, which forms a flexible micro-film that blunts crack tips and impedes oxidation, slowing fatigue evolution, and (ii) the synergism between Synthetic wax and Plasticizer. Synthetic wax raises the elastic modulus at later stages, enhancing deformation resistance, while Plasticizer provides persistent plasticisation that homogenises stress and prevents local concentrations, collectively decelerating damage accumulation and prolonging pavement life.

The VECD model parameters obtained by numerical regression are listed in Table 5. *α* characterises the frequency-dependent response of the storage modulus, whereas *C*_1_ and *C*_2_ quantify the correlation between the loss modulus |G*|sin*δ* and the accumulated fatigue-damage variable *D*(*t*). In this study, fatigue failure is defined as the point at which |G*|sin*δ* decays to 35% of its initial value, from which the characteristic parameters *A*_35_ and *B* are derived. *B* specifically reflects the material’s sensitivity to imposed strain amplitudes.

Table 5 shows that the *α*-values of the different binders are similar, but all decrease monotonically with increasing temperature, confirming the pronounced temperature-dependence of the storage modulus. A higher temperature, therefore, corresponds to a lower *α* and a reduced ability of the material to maintain a high modulus. The *A*_35_ parameter, defined as the accumulated damage when |G*|sin*δ* falls to 35% of its initial value, declines for every binder as the temperature rises. At each test temperature, however, the *A*_35_ of the 11%PWMA is consistently higher than that of the 70^#^, indicating that 11%PWMA can tolerate a greater amount of cumulative damage before fatigue failure occurs. Consequently, 11%PWMA exhibits superior fatigue resistance across the entire temperature range. In addition, the absolute value of *B* decreases with temperature for all samples. For 11%PWMA, this reduction implies a diminished sensitivity to strain amplitude and therefore a slower decay of fatigue life as the temperature increases.

#### 4.5.3. Influence of PNSK on Fatigue Life

**Figure 9:**
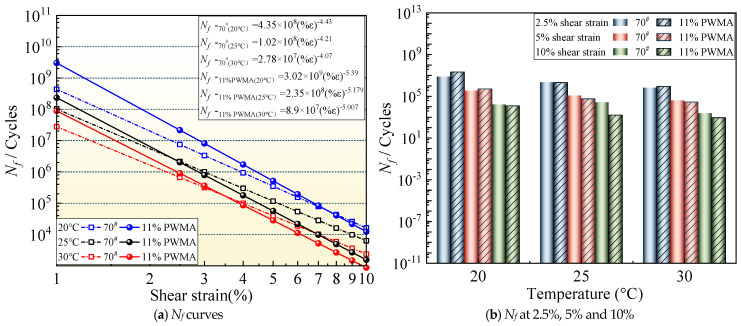
Fatigue life of different asphalt binders at different strain levels and temperatures.

Based on the VECD model calculation method, fatigue equations for 70^#^ and 11%PWMA under varying strain levels were derived, with corresponding fatigue life versus strain curves plotted in Figure 9. Figure 9 further validates the coupled negative effect of temperature and strain amplitude—elevations in both parameters significantly accelerate fatigue damage and shorten pavement service life, a conclusion highly consistent with the aforementioned stress–strain analysis. For quantitative fatigue assessment, VECD-based fatigue life predictions for these two asphalt binders were performed at three typical strain levels (2.5%, 5%, and 10%) representing low, medium, and high stress conditions, with results presented in Figure 9 [45].

Under 2.5% low strain, both asphalt binders exhibit decreasing fatigue life with temperature increase. At 20 °C, 11%PWMA shows significantly higher fatigue life than 70^#^, demonstrating superior resistance to fatigue damage under lower temperature and strain conditions, likely attributable to its three-dimensional reinforcement network that enhances cumulative damage resistance. However, this advantage diminishes progressively at 25 °C and 30 °C, suggesting temperature-induced degradation of 11%PWMA’s internal structure and anti-fatigue properties, though it still maintains marginally better performance than 70^#^. At 5% medium strain, a similar temperature-dependent decreasing trend is observed, with 11%PWMA retaining a fatigue life advantage over 70^#^ at 20 °C, but this reverses at higher temperatures, where 11%PWMA’s fatigue life becomes lower than 70^#^. Under 10% high strain, 11%PWMA experiences greater fatigue life reduction than 70^#^ during 20 °C to 30 °C temperature elevation. Overall, 11%PWMA exhibits a higher fatigue life decay rate than 70^#^, primarily due to its extremely high initial life (3.02 × 10^9^ at 20 °C) decaying to the thousand-level in high-strain regions, indicating greater sensitivity to strain and temperature increases. Notably, WMA generally exhibits relatively high temperature sensitivity as an inherent characteristic of its technical mechanism, representing a genuine engineering challenge that can be controlled within acceptable limits through material optimization and process refinement [46].

### 4.6. High-Temperature Repeated Creep Behavior of the Asphalt Binders

#### 4.6.1. Analysis of the Asphalt Binder’s Creep Behavior

Following AASHTO TP 350-14, repeated-creep loading was applied to 70^#^ and 11%PWMA at high temperatures ranging from 42 °C to 66 °C in 6 °C increments under two stress levels of 0.1 kPa and 3.2 kPa [32,47].

**Figure 10:**
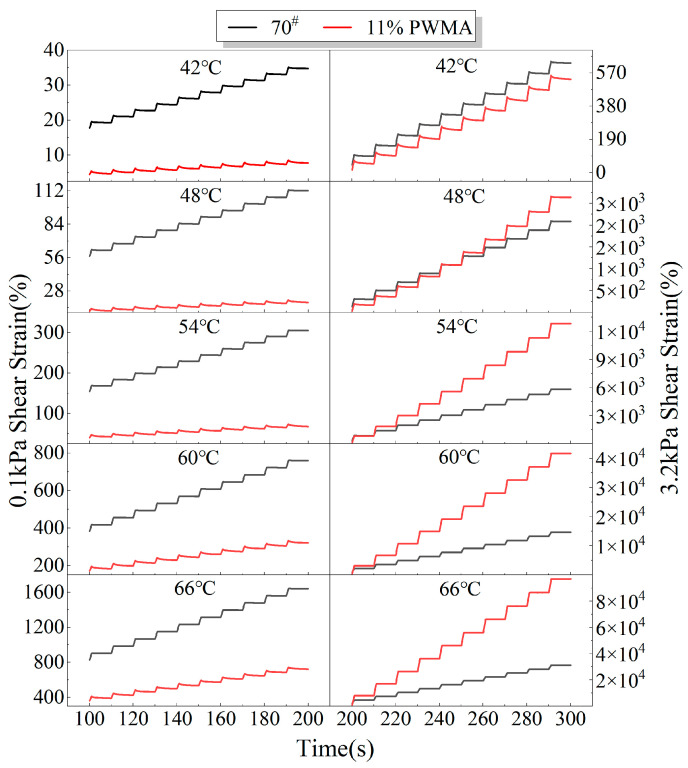
Time-strain curves of different asphalt binders at different temperatures.

Figure 10 presents the creep response of 70^#^ and 11%PWMA under 0.1 kPa and 3.2 kPa at various temperatures. In all cases, the strain increases monotonically with loading time. After each 1 s load/9 s unload cycle, a residual strain remains, and its accumulation becomes more pronounced as the number of cycles grows. Once the cumulative strain exceeds a critical threshold, the material undergoes irreversible failure, closely replicating the in-service mechanical behaviour of asphalt binder pavements [48]. At 0.1 kPa, both binders exhibit small deformations and low growth rates, whereas raising the stress to 3.2 kPa markedly increases the magnitude and rate of strain, confirming that cyclic plastic deformation scales positively with stress intensity [49]. Under the lower stress level, the ranking 70^#^ > 11%PWMA holds across all test temperatures, demonstrating that 11%PWMA possesses superior resistance to external loading and high-temperature deformation compared with the 70^#^.

Under the high-stress level, the accumulated strain of the 11%PWMA remains lower than that of the 70^#^ when the temperature is below 48 °C, indicating retained load-bearing and rutting-resistant capacities. Once the temperature exceeds 48 °C, however, the strain of the 11%PWMA surpasses that of the 70^#^.

#### 4.6.2. Analysis of Creep Recovery Rate and Stress Sensitivity of the Asphalt Binders

**Figure 11:**
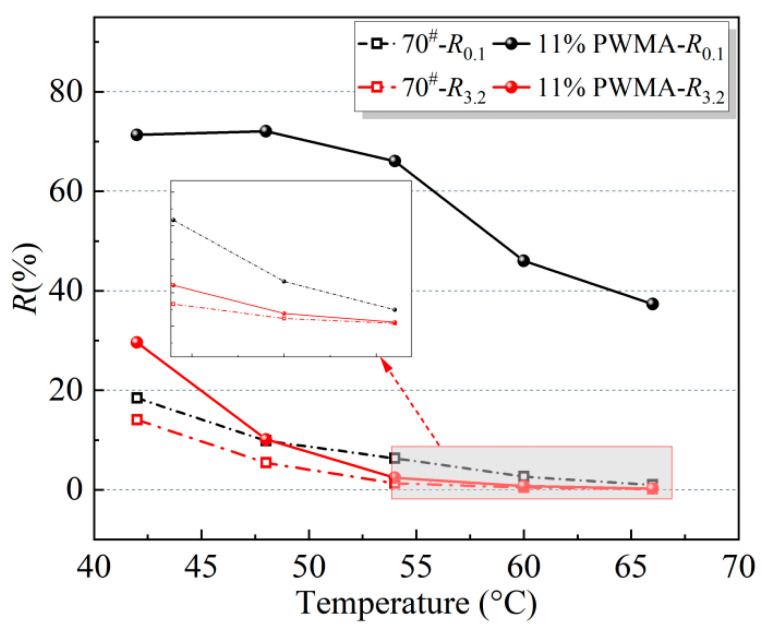
*R* of different asphalt binders at different temperatures.

**Figure 12:**
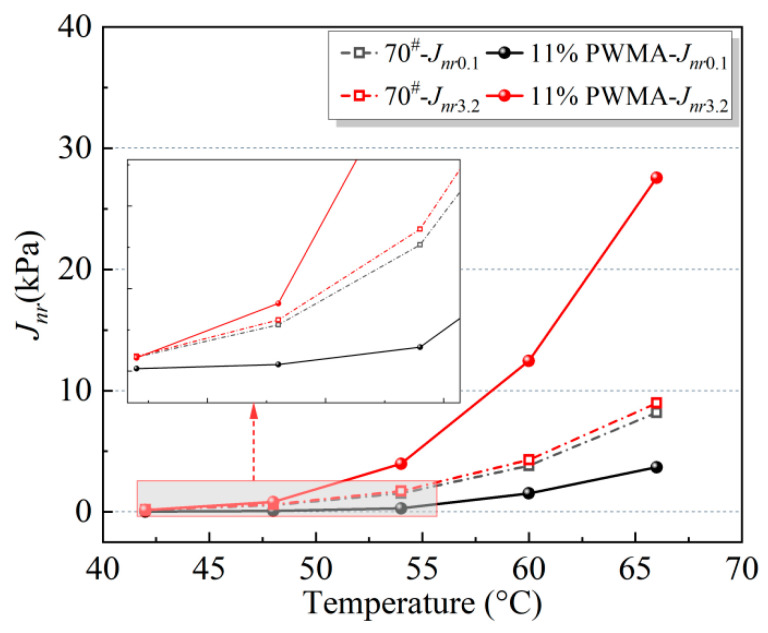
*J_nr_* of different asphalt binders at different temperatures.

**Figure 13:**
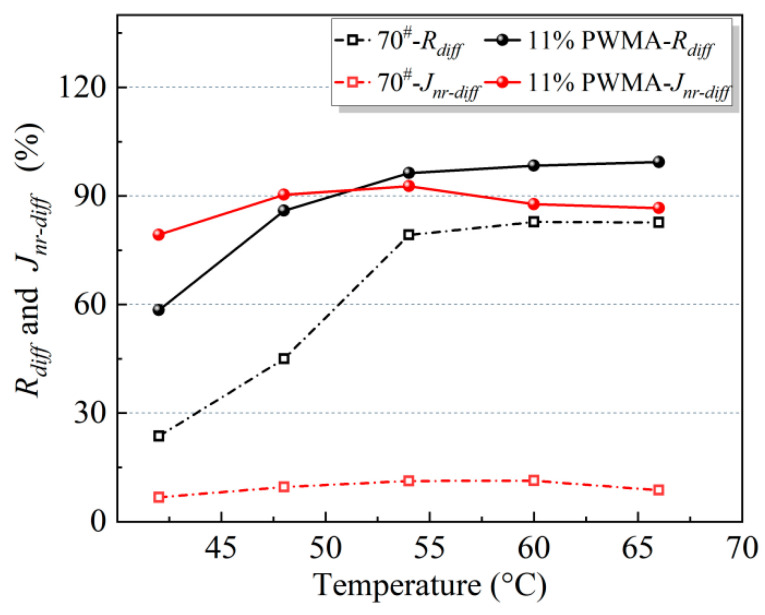
*R_diff_* and *J_nr-diff_* of different asphalt binders at different temperatures.

Based on Equations (13)–(22), the MSCR test indices of the binders were calculated. To clearly present the performance parameters obtained from the MSCR measurements, the results are converted into visual diagrams, as detailed in Figure 11, Figure 12 and Figure 13.

Figure 11 clearly shows that, under both stress levels, the elastic recovery of the binders decreases markedly as the temperature rises from 42 °C to 66 °C, with the mechanical response gradually becoming predominantly viscous. Comparative analysis further reveals that the higher stress level induces a larger amount of non-recoverable deformation in the asphalt materials.

Under 0.1 kPa, the strain-recovery capacity of both binders deteriorates with increasing temperature. However, the WMA consistently exhibits a higher recovery ratio than the 70^#^ across the entire temperature range. Within a specific temperature window the WMA recovery decreases only gradually, whereas beyond this window the decline accelerates markedly. This behaviour indicates that, at low stress levels, WMA can preserve satisfactory elastic recovery and structural stability over a moderate temperature interval. When the stress is raised to 3.2 kPa, recovery ratios drop sharply with temperature. At low temperatures, WMA still outperforms the base binder, demonstrating superior elasticity and a better ability to regain its original shape under high stress. As the temperature rises steadily, the strain recovery rates of both 11%PWMA and 70^#^ decline gradually. Once the critical range is surpassed, the recovery values of both materials approach a low level. However, 11%PWMA retains a marginally superior reserve. Under the coupled effects of elevated temperature and high stress, the elastic characteristics of the 70^#^ fade markedly, whereas 11%PWMA exhibits a more gradual viscoelastic transition. Its structural resilience and resistance to deformation remain slightly ahead, offering the pavement a comparatively mild avenue for stress relief.

The non-recoverable creep compliance (*J_nr_*) of asphalt materials serves as a crucial indicator for characterizing permanent deformation properties, with its magnitude exhibiting an inverse correlation with high-temperature performance. As observed in Figure 12, under both stress levels of 0.1 kPa and 3.2 kPa, the *J_nr_* values of both asphalt binder specimens demonstrate a significant increasing trend as the test temperature rises from 54 °C to 66 °C, reflecting the progressive deterioration of deformation resistance at elevated temperatures. Comparative analysis reveals that when the stress level increases from 0.1 kPa to 3.2 kPa, both 70^#^ and 11%PWMA exhibit marked increases in *J_nr_*, indicating that elevated stress substantially compromises the material’s deformation resistance and adversely affects its high-temperature rheological properties. Under the stress condition of 0.1 kPa, the *J_nr_* of 11%PWMA is lower than that of 70^#^, demonstrating that 11%PWMA exhibits superior resistance to permanent deformation under low-stress conditions, particularly at high temperatures [50]. This is attributed to Synthetic wax in 11%PWMA, which establishes a stable microcrystalline network structure within the binder under typical low-stress conditions (0.1 kPa), significantly enhancing the binder’s elastic recovery capability and deformation resistance. Consequently, the *J_nr_* values are markedly superior to the 70^#^, especially in high-temperature environments. This stress-adaptive characteristic endows 11%PWMA with exceptional durability under conventional traffic load conditions. Simultaneously, Plasticizer and Coupling agent optimize the viscosity-temperature characteristics, ensuring workability while imparting broader temperature adaptability to 11%PWMA, particularly maintaining stable performance at elevated temperatures [51]. Antioxidant compatibilizer enhances the compatibility between the various components and the asphalt binder, as well as storage stability, ensuring lasting performance. This multi-component synergistic design enables WMA to exhibit excellent resistance to permanent deformation under conventional stress conditions and achieve performance optimization across different working conditions through a stress-adaptive mechanism.

Stress sensitivity can be quantified by recovery rate difference (*R_diff_*) and non-recoverable compliance difference (*J_nr-diff_*), where elevated values signify increased sensitivity of asphalt specimens to stress changes [52,53]. Figure 13 reveals that 11%PWMA exhibits higher *R_diff_* and *J_nr-diff_* values than 70^#^ at all temperatures, indicating greater stress sensitivity and more pronounced performance variations under different stress states. This conclusion is highly consistent with the fatigue life analysis discussed above.

## 5. Conclusions

(1) Using 70^#^ as the reference, a four-component PNSK was formulated, and its modified binder was investigated across physical properties and rheological performance. The principal findings are as follows: PNSK reduces asphalt viscosity in a dosage-dependent manner, with cooling benefits leveling off beyond 11%. At this optimum dosage, penetration increases by 28.4%, softening point rises by 30.1%, and ductility improves by 9.4%, indicating balanced improvements in workability, high-temperature stability, and low-temperature cracking resistance. However, adhesion drops sharply when PNSK exceeds 12% (39% reduction at 12%, 67% at 13%). Thus, 11% is the ideal content, balancing all properties.
(2) This study compares the fatigue performance of 70^#^ and 11%PWMA at 20 °C, 25 °C, and 30 °C. The 11%PWMA exhibits a gentler pre-peak slope and a wider peak strain range, indicating lower strain sensitivity and higher strain accommodation capacity. Its fatigue damage progresses gradually, whereas the 70^#^ shows brittle failure after a critical threshold. VECD parameters confirm that 11%PWMA has higher A35 and lower |*B*| at all three temperatures, reflecting reduced strain sensitivity. At 2.5% strain and 20 °C, the fatigue life of 11%PWMA is approximately one order of magnitude higher than that of 70^#^, though this advantage diminishes at 25–30 °C or 5–10% strain.
(3) MSCR tests at 0.1 kPa and 3.2 kPa (42–66 °C) show that under low stress, 11%PWMA has lower accumulated strain and higher recovery (*R*) across all temperatures, confirming excellent permanent deformation resistance under routine traffic. Under high stress (3.2 kPa), its strain remains lower than 70^#^ below 48 °C but approaches or slightly exceeds it above 48 °C, reflecting the temperature sensitivity of warm-mix additives. *R_diff_* and *J_nr-diff_* are higher for 11%PWMA at all temperatures, consistent with fatigue life findings.
(4) A composite warm-mix additive PNSK has been developed, which improves workability, high/low-temp stability, and crack resistance without sacrificing adhesion, filling a gap in balancing rutting and cracking. However, all tests were performed only on the 70^#^ at the binder and binder-aggregate scale. No mixture level tests have been conducted. Therefore, future work will first be carried out on mixture level performance trials, including moisture damage, low temperature cracking, and rutting tests. The application of PNSK should also be extended to polymer-modified asphalts to verify its broader applicability.

## Figures and Tables

**Table 1:** Technical properties of four components.

Properties	Component 1	Component 2	Component 3	Component 4
Appearance	White particles	Pale yellow liquid	Colorless transparent liquid	Colorless transparent liquid
	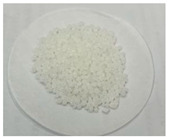	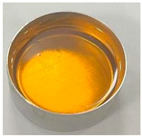	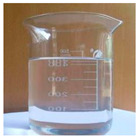	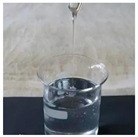
Density (g·cm^−3^)	0.96	0.90	0.95	0.96
Melting point (°C)	92	-	-	-
Solidification point (°C)	-	−38	−50	−59
Refractive index (25 °C)	1.51	1.57	1.43	1.40
Flash point (°C)	270	210	≥230	≥270

**Table 2:** Physical characteristics test results of different asphalt binders.

Type of Asphalt Binders	Penetration (25 °C)/(0.1 mm)	Increase Rate (%)	Softening Point (°C)	Increase Rate (%)	Ductility (10 °C)/(cm)	Increase Rate (%)
70^#^	55.7	0	48.7	0	52.0	0
9%PWMA	70.8	27.1	60.9	25.1	54.4	4.6
10%PWMA	71.7	28.7	61.4	26.1	56.3	8.3
11%PWMA	73.2	31.4	63.3	30.0	56.9	9.4
12%PWMA	74.6	33.9	65.2	33.9	57.3	10.2
13%PWMA	76.1	36.6	66.0	35.5	58.2	11.9

**Table 3:** Specific construction temperatures of different asphalt binders.

Type of Asphalt Binders	Mixing Temperature (°C)	Compaction Temperature (°C)	Temperature Reduction Effect (°C)
70^#^	154~159	144~148	0
9%PWMA	139~144	129~133	15
10%PWMA	137~141	127~131	17
11%PWMA	134~139	125~129	20
12%PWMA	133~138	124~128	21
13%PWMA	130~134	121~125	24

**Table 4:** BBS pull-off test results of different asphalt binders.

Type of Asphalt Binders	Pull-off Strength (MPa)	Decrease Rate (%)
70^#^	3.05	0
9%PWMA	2.15	29.5
10%PWMA	2.10	31.1
11%PWMA	2.04	33.1
12%PWMA	1.24	59.3
13%PWMA	0.99	67.5

**Table 5:** LAS parameters of different asphalt binders at 20 °C, 25 °C and 30 °C.

Type of Asphalt Binders	Temperature (°C)	*α*	*A*_35_	*B*	*C*_1_	*C*_2_
	20 °C	2.210	4.35 × 10^8^	−4.430	0.089	0.507
70^#^	25 °C	2.105	1.02 × 10^8^	−4.210	0.043	0.561
	30 °C	2.035	2.78 × 10^7^	−4.070	0.016	0.681
	20 °C	2.695	3.02 × 10^9^	−5.390	0.182	0.397
11%PWMA	25 °C	2.590	2.35 × 10^8^	−5.179	0.153	0.376
	30 °C	2.553	8.90 × 10^7^	−5.007	0.136	0.364

## Data Availability

The original contributions presented in this study are included in the article. Further inquiries can be directed to the corresponding author.

## Abbreviations

The following abbreviations are used in this manuscript.

Full Term	Abbreviation
A Composite Warm-Mix Additive	PNSK
Base Asphalt Binder	70^#^
Hot-Mix Asphalt	HMA
Linear Amplitude Sweep	LAS
Multiple Stress Creep and Recovery	MSCR
Non-recoverable Creep Compliance	*J_nr_*
PNSK Warm-Mix Asphalt Binder	PWMA
PWMA with a% PNSK	a%PWMA
Strain-Recovery Ratio	*R*
The Binder Bond Strength	BBS
The Viscoelastic Continuum Damage	VECD
The cumulative damage index	*D*(*t*)
The Integrity of the Asphalt Binder Specimen	*C*
The Fatigue-performance Parameter	*N_f_*
The Stress-sensitivity of the Asphalt Binders	*R_diff_* and *J_nr-diff_*
